# Dysfunctional epileptic neuronal circuits and dysmorphic dendritic spines are mitigated by platelet-activating factor receptor antagonism

**DOI:** 10.1038/srep30298

**Published:** 2016-07-22

**Authors:** Alberto E. Musto, Robert F. Rosencrans, Chelsey P. Walker, Surjyadipta Bhattacharjee, Chittalsinh M. Raulji, Ludmila Belayev, Zhide Fang, William C. Gordon, Nicolas G. Bazan

**Affiliations:** 1 Neuroscience Center of Excellence, Louisiana State University Health Sciences Center, 2020 Gravier Street, New Orleans, Louisiana 70112, USA; 2 Department of Pediatrics, Hematology-Oncology, Louisiana State University Health Sciences Center and Children’s Hospital of New Orleans, New Orleans, Louisiana 70118, USA; 3 Biostatistics, School of Public Health, Louisiana State University Health Sciences Center, 2020 Gravier Street, New Orleans, Louisiana 70112, USA

## Abstract

Temporal lobe epilepsy or limbic epilepsy lacks effective therapies due to a void in understanding the cellular and molecular mechanisms that set in motion aberrant neuronal network formations during the course of limbic epileptogenesis (LE). Here we show in *in vivo* rodent models of LE that the phospholipid mediator platelet-activating factor (PAF) increases in LE and that PAF receptor (PAF-r) ablation mitigates its progression. Synthetic PAF-r antagonists, when administered intraperitoneally in LE, re-establish hippocampal dendritic spine density and prevent formation of dysmorphic dendritic spines. Concomitantly, hippocampal interictal spikes, aberrant oscillations, and neuronal hyper-excitability, evaluated 15–16 weeks after LE using multi-array silicon probe electrodes implanted in the dorsal hippocampus, are reduced in PAF-r antagonist-treated mice. We suggest that over-activation of PAF-r signaling induces aberrant neuronal plasticity in LE and leads to chronic dysfunctional neuronal circuitry that mediates epilepsy.

A dynamic neuropathological process underlies the development of mesial temporal lobe epilepsy or limbic epileptogenesis (LE)^1^. Although cellular and molecular inflammatory mechanisms are thought to be involved in LE^2^,^3^,^4^,^5^,^6^,^7^, their significance in promoting homeostasis or exacerbating damage to neuronal network function remains incompletely understood.

Dendritic spines (DS) are critical components of the neuronal network. These spines are vulnerable to pathological plasticity after seizures. DS are protrusions, mostly from dendritic membranes, that contain neurotransmitter receptors and postsynaptic molecular signaling systems^8^, and they receive and integrate excitatory synaptic input from pre-synaptic terminals^9^. DS modulate neuronal excitability and cognitive processes^10^ and are susceptible to seizure-mediated damage^11^, which can in turn lead to abnormal excitability and co-morbidity in epilepsy^12^. Moreover, increased excitatory synaptic activity induces spine formation, but excessive and unrestrained activation can instigate excitotoxicity with DS loss. There is a progressive increase of neuronal hyper-excitability in epileptogenesis^13^,^14^, and modulation of such hyper-excitability could both protect DS and promote neuronal homeostasis after injury.

Platelet-activating factor (PAF, 1-alkyl-2-acetylglycerophosphocholine) is a phospholipid mediator that: a) is synthesized rapidly upon brain stimulation and modulates synaptic plasticity^15^; b) triggers molecular signaling related to neurotransmission^16^ and cellular damage^17^; and c) induces cyclooxygenase-2 (COX-2) gene expression^18^, increasing resultant molecular signaling in post-synaptic areas^19^ after seizures^20^. The extent to which PAF itself is upregulated in the hippocampus after kindling-induced seizures remains unknown – a gap that the current study addresses.

PAF exerts its bioactivity through a G-protein-coupled receptor (PAF receptor; PAF-r)^21^ in synaptic sites^22^. PAF-r is actively engaged in neuroinflammatory signaling associated with brain injuries^23^ such that PAF-r deletion reduces brain damage^24^,^25^,^26^. The PAF-r antagonist LAU-0901 limits kindling epileptogenesis and induces neuroprotection^17^.

In the current study, we show that: a) PAF increases after status epilepticus (SE); b) ablation of PAF-r limits epileptogenesis; and c) bioactivity of PAF-r antagonists reduces seizure susceptibility. Furthermore, our data provide insight into the mechanism of this reduction in seizure susceptibility, specifically showing that PAF-r antagonism protects DS in LE, thus modulating chronic epileptic hippocampal neuronal networks. Our findings suggest that PAF-r activation after brain injury is a key contributor to dysfunctional neuronal circuitry in epileptogenesis and may contribute to limbic seizures.

## Results

### PAF increases after status epilepticus, and the PAF receptor mediates neuronal network hyper-excitability induced by acute seizures

**Figure 1:**
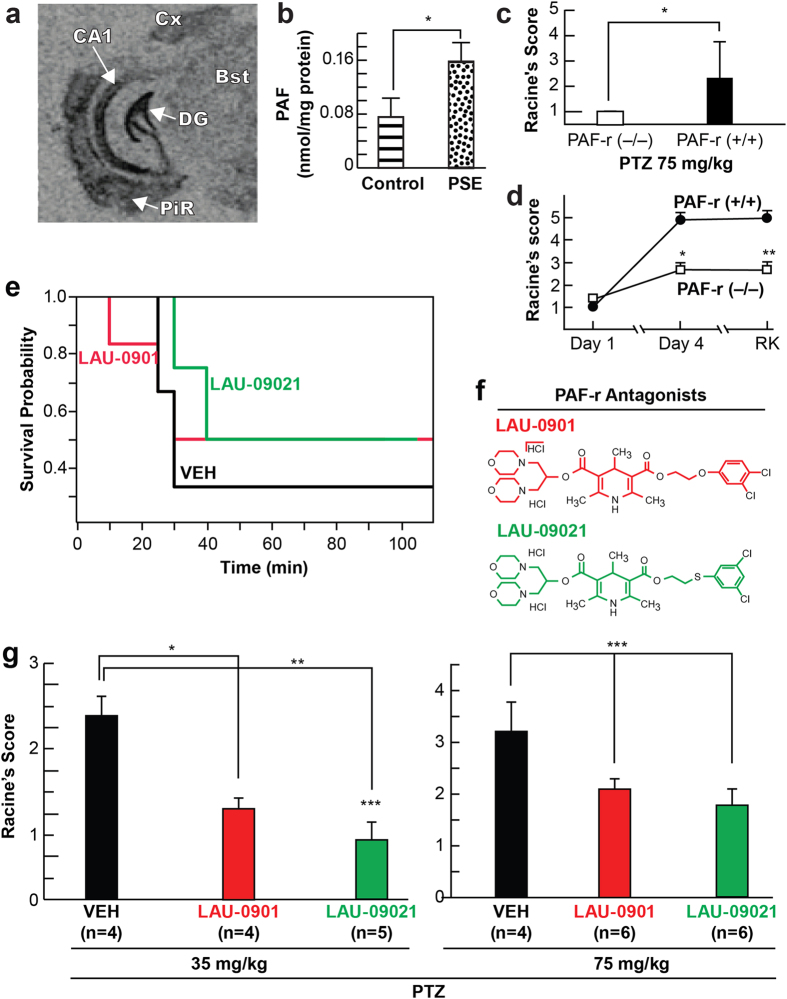
PAF-r antagonist reduces seizure susceptibility. (**a**) Autoradiographic images showing *in situ* hybridization of PAF-r mRNA in the coronal section of an adult naïve mouse half brain. Note high-density signaling, specifically in the dentate gyrus (DG), cornus ammonis 1 (CA1) hippocampal regions and piriform cortex (PiR) compare with cortex (Cx) and brain stem (Bst). (**b**) Hippocampal PAF concentration, measured by LC-MS-MS, increases at 24 hours after termination of pilocarpine-induced status epilepticus (PSE) in mice (n = 3 for both Control and PSE). Bars represent average and S.E.M; *p < 0.05, t-test. (**c**) PAF-r^−/−^ mice are resistant to seizures induced by PTZ; PAF-r^−/−^ (n = 5), PAF-r^+/+^, (n = 6); *p = 0.02 (t-test). (**d**) PAF-r deficient mice (PAF-r^−/−^) limit kindling epileptogenesis. PAF-r^−/−^ (n = 12) mice show attenuation of seizure severity during kindling epileptogenesis (day 1 to day 4) and, as a consequence, seizure susceptibility is limited one week after kindling (rekindling, RK) compared to wild type mice (PAF-r^+/+^, n = 7). (**e**) Kaplan-Myers survival plot from treated mice after PTZ (75 mg/kg), p = 0.8 (**f**) Molecular structure of PAF-r antagonists. Note sulfur (S) as a heteroatom in the new PAF-r antagonist LAU-09021. (**g**) Administration of PAF-r antagonists LAU-0901 and LAU-09021 reduce seizure severity induced by PTZ at sub-convulsive (35 mg/kg) and convulsive (75 mg/kg) doses respectively. PAF-r antagonists or vehicle were administered intraperitoneally two hours before PTZ injection. Dots and squares indicate means. *p:0.0034; **p: 0.011, t-test. Bars indicate means, and error bars represent S.E.M. *p < 0.001; **p < 0.0001, ***p = 0.03; ANOVA).

Limbic epileptogenesis starts at the termination of SE (post-status epilepticus; PSE) in a rodent model of temporal lobe epilepsy (TLE)^27^. Thus, to test the potential significance of PAF receptors during LE, we first detected PAF-r expression using *in situ* hybridization. This revealed that PAF-r were localized in the limbic area, preferentially in the dentate gyrus (DG) and CA1 regions of the hippocampus (Fig. 1a). Next, to test the hypothesis that PAF increases after SE, we used liquid chromatography tandem mass spectrometry (LC-MS-MS) analysis and found that PAF increased in hippocampi 24 hours after SE (naïve: 0.07 vs PSE: 0.15 nM/mg protein; p < 0.05; Fig. 1b). Using the pentylenetetrazol (PTZ) test (see Methods), we asked if PAF-r mediates seizure susceptibility by comparing seizure severity between male mice deficient in PAF-r (*PAF-r*^−/−^)^28^ and their age-matched wild type mice (*PAF-r*^+/+^) following PTZ administration at the convulsive dose (75 mg/kg). We observed that *PAF-r*^−/−^ is resistant to induced acute tonic-clonic seizures (Fig. 1c, Racine score: *PAF-r*^−/−^ 1.0 ± 00 vs. *PAF-r*^+/+^ *2.3* ± 1.4, p = 0.02), requiring 2.4 times more PTZ (185 mg/kg) to induce seizures (Supplementary Fig. 1). Then, using a rapid kindling model of LE^13^, we compared progression of seizures in adult male mice deficient in PAF-r (*PAF-r*^−/−^)^28^ and their age-matched wild type mice (*PAF-r*^+/+^), uncovering a remarkable attenuation of seizure severity at day 4 (Fig. 1d Racine score: *PAF-r*^−/−^ 2.81 ± 0.42 vs. *PAF-r*^+/+^ 4.74 ± 0.12, p = 0.0034). Additionally, seizure susceptibility was limited in PAF-r^−/−^ mice one week after kindling (rekindling, RK) (Fig. 1d, Racine score: *PAF-r*^−/−^ 2.8 ± 0.5 vs. *PAF-r*^+/+^ 4.71 ± 0.28, p = 0.011).

We showed previously that the PAF-r antagonist LAU-0901 reduces progression of seizure severity and seizure susceptibility in models of temporal lobe epilepsy^17^. Here, we studied the bioactivity of a newly formulated PAF-r antagonist, LAU-09021, against seizures using the PTZ test on a screened series of PAF-r antagonist compounds (Supplementary Fig. 2a,b). LAU-09021 has a sulfur molecule as a heteroatom (Fig. 1f and Supplementary Fig. 2b). We observed that LAU-09021 reduced partial limbic and generalized seizures compared to the vehicle, and was more efficient than LAU-0901 at reducing seizures (Fig. 1g). Moreover, LAU-09021-treated mice displayed an increased survival rate after receiving a convulsive dose of PTZ, as compared to vehicle-treated mice (Fig. 1e).

### PAF-r antagonism restores DS integrity and reduces seizure susceptibility in epileptogenesis

**Figure 2:**
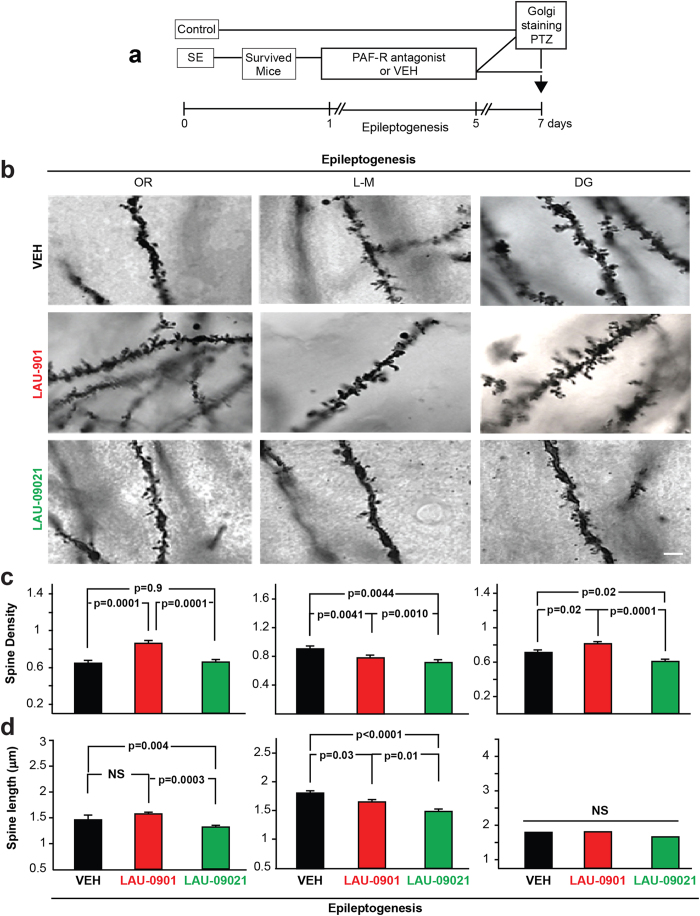
PAF-r antagonism restores dendritic spine density in epileptogenesis. (**a**) Representative experimental design. (**b**) Golgi staining showing high magnification of representative dendrites from OR (stratum oriens), L-M (stratum lacunosum-moleculare) and DG (outer molecular layer of dentate gyrus) hippocampal layers from coronal section (40 μm) of the dorsal hippocampus from vehicle (VEH, n = 5), LAU-0901- (n = 6) and LAU-09021- (n = 6) treated animals at 7 days after SE (Epileptogenesis). (**c**) Average spine density per dendrite segment. Note that LAU-0901-treated mice have higher density compared to LAU-09021- and vehicle-treated mice. (**d**) Average spine length per dendrite segment showing an increase in LAU-0901-treated mice in the OR and decreased in L-M compared to vehicle. Note LAU-09021-treated mice have a reduction of dendritic spine length in OR and L-M. Bars indicate means, and error bars represent S.E.M.; p = p values, ANOVA. Scale bar: 10 μm.

Spines are damaged after repeated seizures and in epilepsy^27^. Here, we tested the hypothesis that the administration of PAF-r antagonists during LE provides protection for DS. LAU-0901 preserves DS density in the stratum oriens (OR) (LAU-0901: 0.8 ± 0.02 vs. vehicle (VEH): 0.65 ± 0.02; p = 0.0001; LAU-09021: 0.65 ± 0.02) and the DG (LAU-0901: 0.8 ± 0.03 vs. VEH: 0.7 ± 0.02; p = 0.02, LAU-09021: 0.60 ± 0.03 Fig. 2a–c). As compared to LAU-09021-treated mice, LAU-0901-treated mice showed an increase in DS length in the OR (LAU-0901: 1.5 ± 0.04 vs. LAU-09021: 1.33 ± 0.04; p = 0.0003) and stratum lacunosum-moleculare (L-M) (LAU-0901: 1.6 ± 0.04 vs. LAU-09021: 1.4 ± 0.04; p = 0.01; Fig. 2b,d). In the DG, neither LAU-0901 nor LAU-09021-treated mice differed from vehicle-treated mice, whereas in the OR, LAU-09021-treated mice showed shorter dendritic spines, as compared to vehicle-treated mice (LAU-09021: 1.3 ± 0.03 vs. VEH: 1.5 ± 0.08; p = 0.004). Alternatively, in the LM, PAF-r antagonism decreased DS length (LAU-0901: 1.6 ± 0.04 vs. VEH: 1.7 ± 0.04; p = 0.03; LAU-09021: 1.4 ± 0.04 vs VEH, p < 0.0001 Fig. 2b,d).

**Figure 3:**
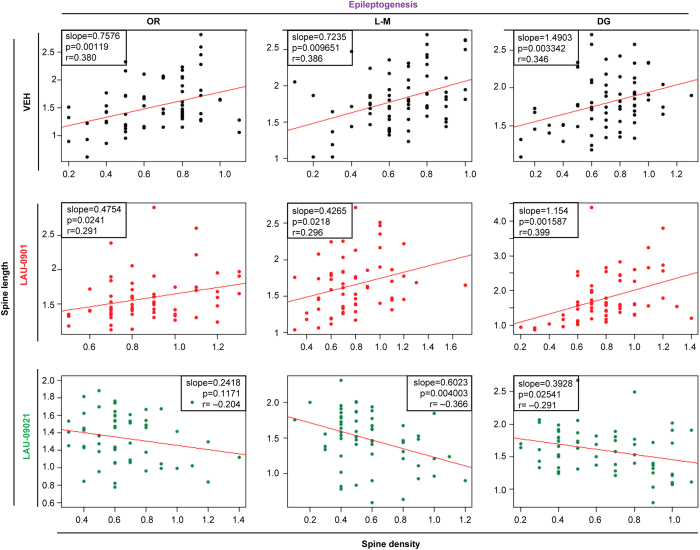
PAF-r antagonism promotes recovery of dendritic spines in epileptogenesis. Correlation analysis describing the relation between spine density and spine length from hippocampal dendrite projections from Vehicle- (VEH, n = 5), LAU-0901- (n = 6) and LAU-09021- (n = 6) treated animals at 7 days after pilocarpine-induced post-SE (Epileptogenesis). Dendritic spine density is positively correlated with length of dendritic spines in vehicle-treated mice and a negative correlation is noticed in LAU-09021-treated animals. OR: stratum oriens; DG: outer molecular layer of dentate gyrus. Slope, p values, and Pearson’s correlation are placed inside of each graph.

**Figure 4:**
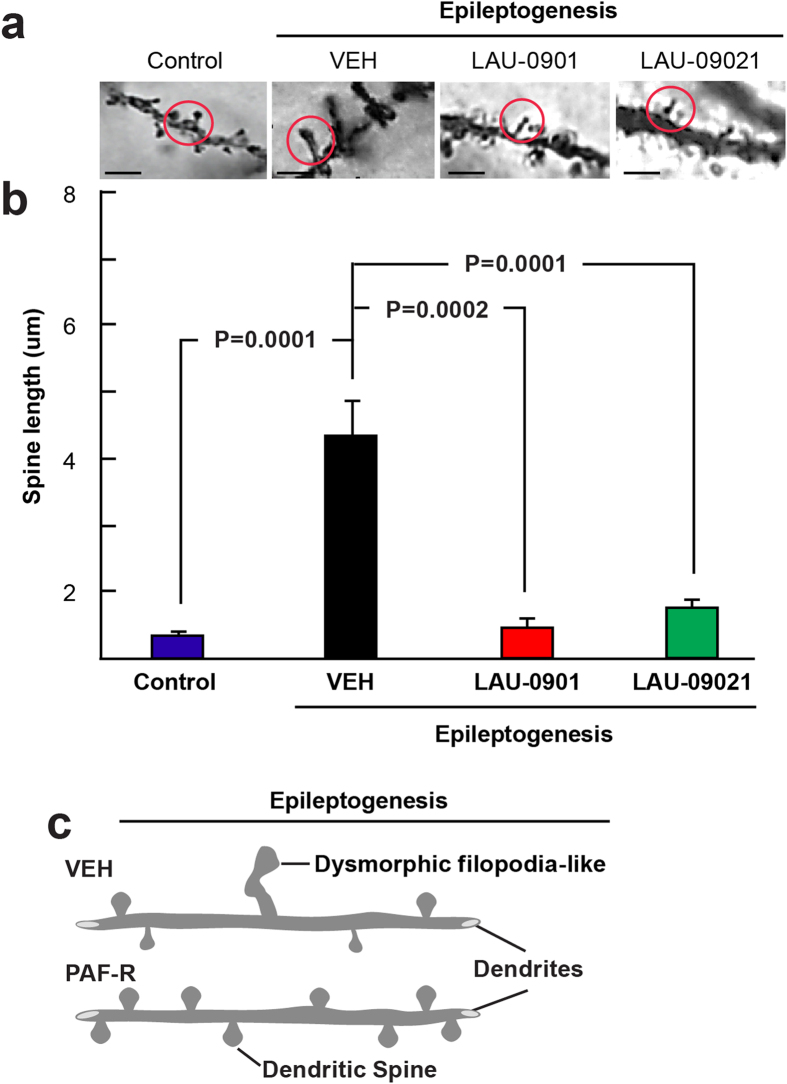
PAF-r antagonism prevents dysmorphic filopodia-like projections in epileptogenesis. (**a**) Golgi staining showing high magnification of dysmorphic filopodia-like spines (red circles) from vehicle-treated animals (VEH, n = 5), and LAU-0901- and LAU-09021-treated animals (n = 6 for both) at 7 days after pilocarpine-induced post status epilepticus (Epileptogenesis). Scale bars indicate 5 μm. Below: Average spine length for dysmorphic filopodia-like spines increases in vehicle-treated mice compared to LAU-0901- and LAU-09021-treated mice. (**c**) A diagrammatic representation of dysmorphic filopodia-like dendritic spines in vehicle- and PAF-r antagonist-treated mice after PSE. Bars indicate means, and error bars represent S.E.M.; p = p values, ANOVA.

**Figure 5:**
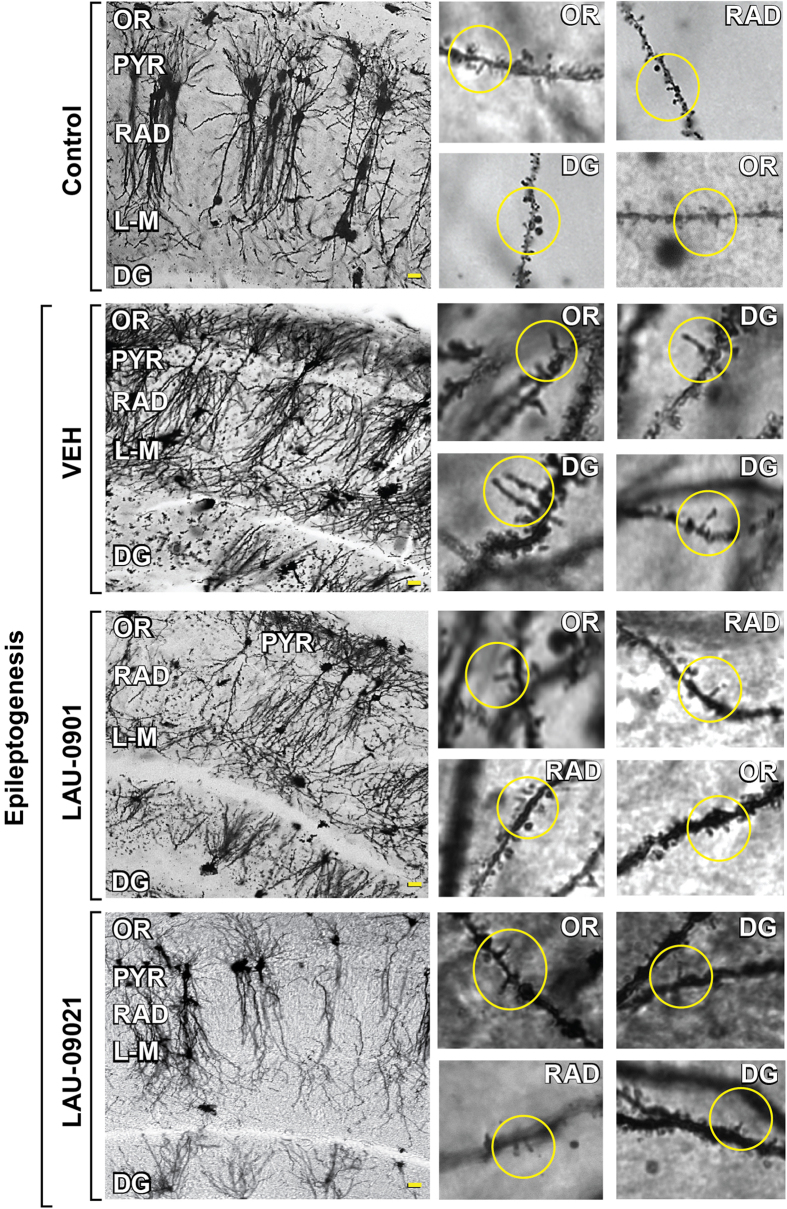
Dysmorphic dendritic spines in epileptogenesis. Left column: Representative hippocampal layers (stratum oriens: OR; pyramidal layer; PYR; stratum radiatum: RAD; stratum lacusosum-moleculare (L-M) and dentate gyrus: DG) from coronal sections (40 μm) of dorsal hippocampus following Golgi staining protocols from control mouse and VEH and PAF-r antagonist compound-treated mice. Bar indicates 10 μm. Right column: representative filopodia and filopodia-like dendritic spine projections (inside circles) different hippocampal regions of control and treated mice respectively. Bars indicate 5 μm.

To analyze the effect of PAF-r antagonism on overall spine integrity, a correlation analysis was performed between the spine density and spine length of PAF-r antagonist-treated animals (Fig. 3). Dendritic spine density correlated positively with DS length in the OR, L-M and DG in LAU-0901-treated mice and vehicle-treated mice, such that lower spine density was associated with shorter spine length. This linearly-positive relation is justified by the fact that the slope of each regression line in the top and middle panels of Fig. 3 is significantly greater than 0 (every corresponding p value is <0.05). Note that the Pearson Correlation Coefficients (VEH: OR *r* = 0.380 LM *r* = 0.386 DG *r* = 0.346 and LAU-0901: OR *r* = 0.291 LM *r* = 0.296 DG *r* = 0.399) indicate moderate correlations, by the rule of thumb, in large data sets. Conversely, DS density correlated negatively with DS length in LAU-09021-treated mice, such that higher densities were associated with shorter spine lengths, with the slopes of regression lines in the LM and DG being significantly less than zero (p < 0.05) but not significant in the OR (p = 0.117; Correlation Coefficients, OR *r* = −0.204 LM *r* = −0.366 DG *r* = −0.291). In addition, we observed that dysmorphic filopodia-like formations from dendrites in vehicle-treated mice were prevented by LAU-0901 and LAU-09021 treatment (VEH: 4.36 ± 0.5; LAU-0901: 0.92 ± 0.2; LAU-09021: 1.46 ± 0.4; p < 0.0001 and 0.0002 respectively with VEH) (Figs 4a–c and 5).

### LAU-09021 lessens seizure onset and hyper-excitability

**Figure 6:**
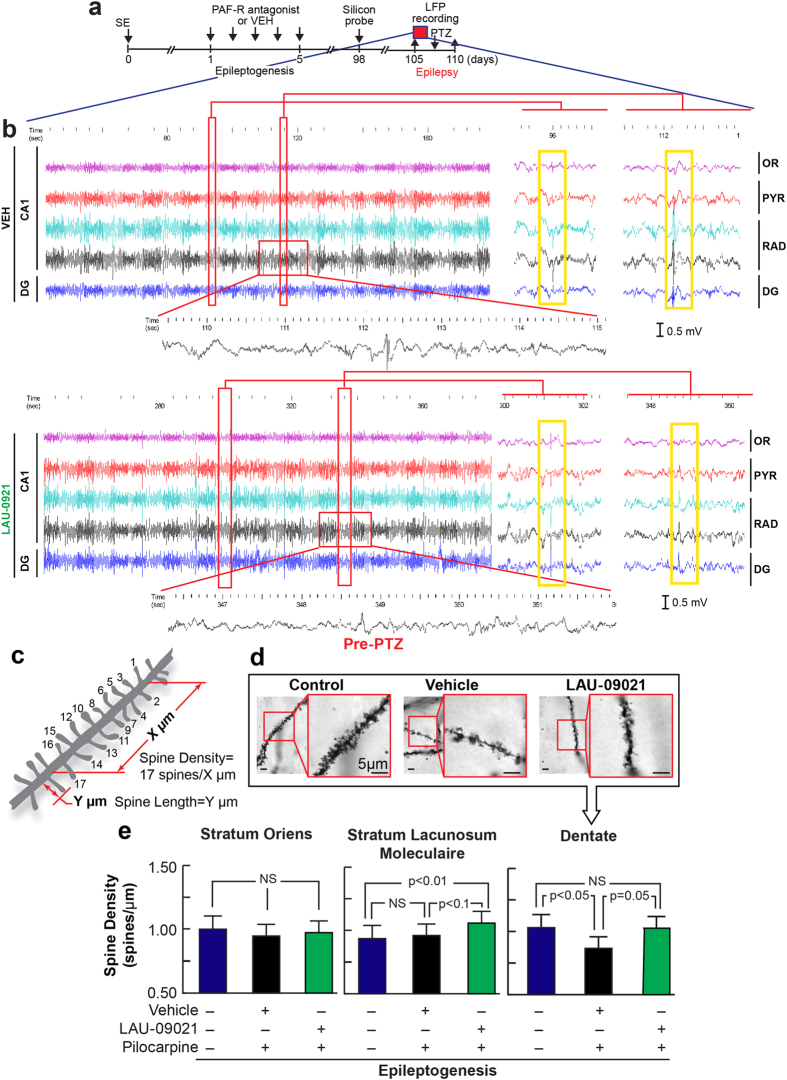
LAU-09021 administration attenuates spontaneous epileptiform activities and dendritic spine changes in the hippocampus during epileptogenesis. (**a**) Experimental design to evaluate spontantous hippocapampal local field potentials (LFP’s) 105 days after SE in LAU-09021- or vehicle (VEH)-treated mice. (**b**) LFP from hippocampal CA1 region: stratum oriens (OR), pyramidal layer (PYR), stratum radiatum (RAD) and dentate gyrus (DG) at multiple time scales. In VEH- treated animals, high amplitude inter-ictal spikes, predominant in RAD, propagate to other sub-fields; these spikes are reduced in LAU-09021-treated mice. (**c**) Schematic indicating spine density measurement. (**d**) Golgi stained dendritic spines of non-epilepsy, epilepsy control-treated, and epilepsy LAU-treated mice (scale bar indicates 5 μm (**e**) Spine density (avg. ± SEM) in various hippocampal regions at 105 days post-SE. In the dentate, vehicle-treated animals show reduced spine density as compared to healthy controls. LAU-09021 restores spine density to the levels observed in healthy controls. In the stratum lacunosum moleculaire, a modest but statistically significant increase in spine density is observed in LAU-09021-treated animals, as compared to vehicle-treated and healthy controls. No differences are observed in the stratum oriens.

Limbic epilepsy is commonly characterized by a high frequency-hyper-excitable limbic neuronal network^30^. Also, hippocampal hyper-excitability and seizures contribute to the progression of the epileptic condition^13^,^31^,^32^,^33^. Our model of TLE reproduces chronic limbic recurrent spontaneous seizures^34^. We tested our hypothesis that LAU-09021 counteracts the chronic hippocampal hyper-excitable state as a consequence of PAF in LE. We characterized hyper-excitable neuronal networks in the hippocampus 15–16 weeks after SE using multi-array microelectrodes inserted into the dorsal hippocampi of freely-moving mice (Fig. 6a and Supplementary Fig. 3). LAU-09021, administered during five consecutive days after SE (epileptogenesis), limited high amplitude spontaneous epileptiform spikes, mainly in the stratum radiatum (RAD) 105 days after SE (Fig. 6b).

At 105 days post-SE, animals were euthanized for spine density (Fig. 6c–e) and length (Supplementary Fig. 4) measurements. At this time point, vehicle-treated animals continue to evidence a decrease in dentate gyrus spine density as compared to healthy controls. This loss in spine density appears to be attenuated in LAU-treated animals. These changes, at the major input region of the hippocampus, may aid in explaining the reduction in epileptiform spikes observed at this time point (additionally, LAU-09021 appears to modestly increase spine density in the stratum lacunosum moleculaire; no effects at 105 days were observed in the stratum oriens).

**Figure 7:**
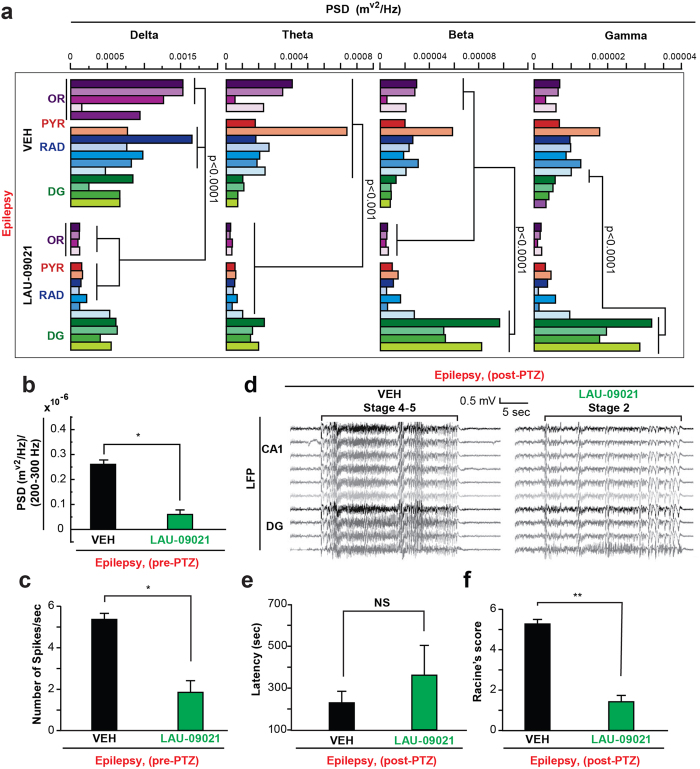
Administration of LAU-09021 during epileptogenesis attenuates chronic hippocampal neuronal network dysfunction. (**a**) Frequency analysis from spontaneous hippocampal LFP 105–110 days after pilocarpine-induced post status epilepticus (Epilepsy) from freely moving vehicle- and LAU-09021-treated mice. Note that LAU-09021 reduces delta and theta waves in the CA1 hippocampal region and increases beta and gamma waves in the dentate gyrus. Each column represents an individual microelectrode from the CA1 stratum oriens (OR), pyramidal layer (PYR), stratum radiatum (RAD) and dentate gyrus (DG). (**b**) High frequency activity (>250 Hz) is reduced in LAU-09021-treated mice compared to vehicle (VEH). (**c**) Number of high amplitude interictal spikes before PTZ are reduced in LAU-09021-treated mice. (**d**) Representative local field potentials of the dorsal hippocampus 110 days after SE (Epilepsy) from vehicle- and LAU-09021-treated mice showing maximal generalized discharge (below Bracket). Note that LAU-09021 reduces hippocampal hyper-excitability and seizure susceptibility. (**e**) LAU-09021 shows a trend of increased latency following PTZ. (**f**) Racine’s score is lower in LAU-09021-treated mice (n = 4) vs. Vehicle (n = 4) after PTZ administration. Vehicle or LAU-09021 (60 mg/kg) was administered only during 5 consecutive days after 24 hours from termination of SE induced by pilocarpine. Bars indicate means, and error bars represent S.E.M. *p < 0.006; t-test. CA1: cornu ammonis; OR: stratum oriens; PYR: stratum pyramidal; RAD: stratum radiatum; DG: dentate gyrus; PSD: power spectral density; LFP: local field potential. mV: millivolts; sec: seconds.

Treatment with LAU-09021 during epileptogenesis reduced the spontaneous power of delta and theta waves in the OR, pyramidal (PYR), and stratum radiatum (RAD) layers; reduced power of beta waves in the OR, increased the power of beta and gamma waves in the DG (Fig. 7a); and reduced high frequency oscillations (HFOs; LAU-09021: 0.5 × 10^−7^ ; VEH: 0.26 × 10^−6^; p < 0.0001) in the DG (Fig. 7b) of the dorsal hippocampus 110 days after SE. Also, hippocampal spontaneous epileptiform spikes were reduced in LAU-09021-treated mice (LAU-09021: 1.8 ± 0.5; VEH: 5.3 ± 0.3, p:0.01) (Fig. 7c). Since neuronal hyper-excitability is a component of the altered neuronal network in epilepsy, we evaluated the degree of induced hyper-excitability following the PTZ test at sub-convulsive doses (Fig. 6d–f, Supplementary Fig. 3). LAU-09021-treated mice displayed a trend of higher latency for seizures compared to vehicle-treated mice (Fig. 6e) and showed reduced seizure severity (Racine’s score: LAU-09021: 1.4 ± 0.7; VEH: 5.2 ± 0.7, p < 0.0001) associated with attenuation of electrical discharges in all hippocampal layers (Fig. 7e,g).

## Discussion

PAF accumulation in the brain after seizures^16^,^24^ or SE (Fig. 1) could sustain glutamate release^35^ and activation of COX-2 gene expression^36^, inducing production of prostaglandin E2 (PGE2)^37^. PGE2 regulates neuronal membrane excitability^38^, which synergistically boosts intraneuronal calcium mobilization mediated by PAF^39^ and facilitates a hyper-excitable state in the neuronal network during epileptogenesis. Therefore, anti-epileptogenesis mechanisms could be mediated by PAF-r antagonism, indicating a novel alternative therapeutic approach to modulating the COX-2 signaling cascade^40^.

Control of the input and output of the hippocampal network^41^ takes place during the functional activation of DS and somatic inhibition^42^,^43^. Also, DS formation is widely assumed to reflect structural reorganization of synaptic connections^44^. Dendritic spines, sites for excitatory synaptic transmission, play a major role in neuronal plasticity in epilepsy. Models of TLE show DS loss^11^,^29^ and increased dendritic length in pyramidal cells^45^, suggesting a cellular mechanism of recovery for increasing synaptic contacts. This is important in maintaining homeostatic synaptic function or formation of abnormal structural reorganization that promotes circuitry reorganization and epileptogenesis. Reduced spine density is associated with reduction of spine length and has been observed in other disorders^46^,^47^. Here, we observed a positive correlation between DS density and length in vehicle and LAU-0901-treated mice, suggesting plasticity as a consequence of SE (Fig. 3). Since LAU-0901-treated animals display greater spine density than vehicle-treated mice (in the OR and DG), LAU-0901 could promote faster recovery of dendritic spines in those hippocampal regions critical for hippocampal connectivity with other brain circuits. Interestingly, a negative correlation between DS density and length was observed in LAU-09021-treated mice. We hypothesize that this scenario reflects use of an alternative homeostatic plasticity mechanism, potentially reflecting different timescales of recovery associated with different pharmacokinetics of the two LAU compounds, a topic for further research.

PAF-r antagonism may elicit protection of neuronal circuitry^48^ in epileptogenesis by increasing post-synaptic contacts in the DG (Fig. 6e). This could improve input from the entorhinal cortex, which may be the origin of the observed increase in DG neuronal network activity (Figs 6b and 7a) and retraction of post-synaptic contact in the stratum lacunosum-moleculare. Such an effect would limit CA3 input from Schaffer collaterals, as reflected by depressing neuronal network activity in the stratum radiatum (Fig. 7a). As a result of this restructuring of the hippocampal circuitry, PAF-r antagonism attenuates onset and propagation of epileptiform activity in the CA1 hippocampal activity (Figs 6b and 7a). On the other hand, LAU-09021 initially facilitates spine recovery, increasing the length rather than the number of spines at 5 days PSE. At 105 days PSE, however, variance in spine length appears diminished, whereas small but significant changes in spine density are still observed. Although a percentage of the observed differences are small in absolute terms, there may be synergistic effects that represent a cumulative benefit. Furthermore, while the biological significance of dendritic spine density and morphometry is well established, it is likely that these parameters vary over a small range such that epileptogenesis cannot instigate total DS loss, inherently limiting the signal window for PAF-r antagonist rescue. However, based on the current observations, we hypothesize that during epileptogenesis there is a dynamic restructuring of dendritic spines that could involve different waves of cellular mechanisms for mediating growth of dendritic spines (LAU-09021) as well as spinogenesis (LAU-0901). If epileptogenesis is interrupted by treatment, the long term cellular strategy of homeostatic plasticity may shift to spinogenesis (as compared to spine growth), as we observed at 105 days post-status epilepticus. Further studies at different time points in epileptogenesis will be required to determine if PAF-r antagonism mediates such dynamic changes in dendritic spines.

Unexpectedly, we observed aberrant filopodia-like spines in vehicle-treated mice (Fig. 4a); these spines were prevented by PAF-r antagonism in the LAU-09021- and LAU-0901-treated mice. At present there are no scientific reports showing that such aberrant dendritic spines occur in epileptogenesis; however, spine alterations are present in developmental and mental disorders^49^,^50^. The structural variation in spines observed in our studies could contribute to the aberrant formation of circuitry and co-morbidities of epilepsy, in addition to disruptive behavior and cognitive deficits. Moreover, prevention of the formation of these aberrant spines by PAF-r antagonism could be involved in reduction of seizure susceptibility and hippocampal hyperexcitability^17^ (Figs 6 and 7). These results provide anatomical and electrophysiological evidence that aberrant dendritic spine formation in epileptogenesis could have an impact on late excitability of neuronal circuitry.

Spontaneous epileptiform events^51^,^52^,^53^,^54^,^55^,^56^,^57^ are associated with brain hyper-excitability^58^,^59^ and reflect aberrant neuronal projections present in TLE^32^. Delta-band activity (0–4 Hz) is characterized by slow waves during slow-wave sleep and drowsy brain states. The elevation of slow-wave activity, especially delta-band activity, is correlated with behavioral changes in simple-partial and complex-partial seizures^60^,^61^. Large amplitude (1–2 Hz) slow activity can also occur in the frontal and parietal neocortices during (ictal) and immediately following (postictal) temporal lobe seizures^61^. Power spectral densities for delta and theta waves were significantly lower in LAU-09021-treated mice three months after SE. This may signify that formation of an aberrant neuronal network in epileptogenesis is limited by PAF-r antagonism and, as a consequence, decreases the establishment of epileptic neuronal circuitry.

High-frequency oscillations at >100 Hz have been recorded in cortical structures of humans and other animals, both under physiological conditions and during partial epilepsies^62^. Intracranial electroencephalography recordings obtained from pharmaco-resistant patients suffering from TLE have shown that HFOs are observed in tandem with an interictal spike^63^,^64^. LAU-09021 significantly reduced HFOs mainly in the DG, suggesting that LAU-09021-treated animals experience attenuation of hippocampal recurrent neuronal circuitry^65^.

Furthermore, beta and gamma waves are depressed after seizures^66^. We observed that LAU-09021 intervention (five days PSE) reduced spontaneous epileptiform activity. Beta and gamma oscillations in the hippocampal CA3 area are modulated by aberrant GABA-mediated neurotransmission from the DG^66^. LAU-09021-treated mice had an increase in beta and gamma oscillations in the DG compared to vehicle-treated animals. The possibility that LAU-09021 could facilitate GABAergic transmission from the DG to the CA3^67^ after seizures *in viv*o should be explored more in depth.

## Conclusion

We conclude that an increase in PAF activates PAF-r in the hippocampus during epileptogenesis, thus mediating neuronal network hyper-excitability and seizure susceptibility. By blocking PAF-r and using the PAF-r antagonist during epileptogenesis, aberrant connectivity in the hippocampus was limited and, as a consequence, onset of epilepsy was reduced. We speculate that PAF-r activity could mediate aberrant connectivity in epileptogenesis.

Taken together, our observations suggest that the neuronal circuitry in the epileptic brain^41^ is enhanced by PAF-r over-activity during epileptogenesis. More experimental studies need to be conducted to elucidate the molecular and neurotransmission-related mechanisms involved in this process. Furthermore, understanding PAF antagonism and the potential therapeutic usefulness of PAF receptor antagonists is relevant to developing disease-modifying therapeutic interventions for patients at risk for epilepsy.

## Methods

### Animals

Studies were performed according to National Institutes of Health guidelines and in accordance with nationally accepted principles in the care and use of experimental animals. The Institutional Animal Care and Use Committee (IACUC) at the Louisiana State University Health Sciences Center (LSUHSC), New Orleans, approved the animal protocols used for this study. Water and food were available for ad libitum consumption. All efforts were made to minimize pain and suffering and to reduce the number of mice used in these experiments. For euthanasia, animals were deeply anesthetized with ketamine hydrochloride and xylazine (200 mg/kg + 10 mg/kg; i.p.) prior to death by decapitation.

A total of 122 C57BL/6 adult male mice (20–25 g; Charles River Labs, Wilmington, MA) and 12 adult male PAF-r (^−/−^) and 5 wild type were used in this study. For PAF-r^−/−^: Donor strain: 129P2/OlaHsd via E14.1 ES cell line, genotype a/aB/BC/C. The mice were developed by Dr. Satoshi Ishii (University of Tokyo, 1998). A neomycin cassette was inserted into the open reading frame of the Ptafr gene. The mutant mice were backcrossed to C57BL/6. Colony maintenance was conducted at the LSUHSC-Neuroscience Center of Excellence and backcrossed to C57BL/6 (Heterozygote x C57BL/6NCrlCrlj). PAF-r^−/−^ mice were bred in-house in our animal facility. For this study, mice were anesthetized, tailed and genotyped as described below. Briefly, genomic DNA preparation was performed by digesting 5 mm tail tips in a mixture of proteinase K and DirectPCR (Tail) reagent (Viagen, Los Angeles, CA) following the manufacturer’s directions. The lysates were spun down and 2 μl of each genomic preparation were used for PCR analysis. PCR were carried out using illustra PuReTaq Ready-To-Go PCR beads (GE Healthcare, Buckinghamshire, UK) with the following conditions: 94 °C 180 s (1 cycle); 94 °C 30 s, 56 °C 30 s, 72 °C 60 s (30 cycles) and, 72 °C 300 s (1 cycle). Primers used for the analysis were as follows: mPAFR Forward 5′-CTCCCACTGTGGATTGTCTACTACT-3′; mPAFR Reverse 5′-AAGATAAGGAAGAAGACGAGGAAGA-3′; Neo CASSETTE 5′-CTATCAGGACATAGCGTTGGCTAC-3′. The combination of the primers used to detect wild-type allele were mPAFR Forward and Reverse, and for the PAF-r^−/−^ allele was mPAFR Forward and Neo CASSETTE. PCR for both alleles was run in separate reactions retrieving a band of approximately 400 bp for WT and 1000 bp for Mutant revealed in a 1% agarose gel stained with Ethidium Bromide.

### Pentylenetetrazol test

Systemic administration of pentylenetetrazol (PTZ), a model used to test potential anti-convulsive effects^27^, was used as an initial step to further evaluate the spectrum of activity of PAF-r antagonist compounds. Based on our preliminary experiments, we observed that: a) intraperitoneal (i.p.) administration of PTZ at 35 mg/kg is useful as a sub-convulsive dose to evaluate non-convulsive seizures, and b) the PAF-r antagonist LAU-0901 has a physiological effect on the brain for 4–6 hours after injection and has no apparent adverse effects^17^,^68^. Animals were placed in individual Plexiglass cages (28 × 28 × 37.5 cm) and given a single dose of PTZ (Sigma, St Louis, MO) at 35 mg/kg/i.p. or 70 mg/kg/i.p. to evaluate effectiveness of the PAF-r antagonist compounds against seizures or to determine chronic hippocampal hyper-excitability and seizure susceptibility as a consequence of LE. Animal recordings (90-minutes in length) were made using a video-recording system (Handycam Sony); recordings began immediately after PTZ injection. At the end of the experiment, animals were euthanized. Locomotor seizures were quantified according to a modified Racine’s Score^69^, as follows: 0, normal behavior—walking, exploring, sniffing, grooming; 1, immobile, staring, jumpy, curled-up posture; 2, automatisms—repetitive blinking, chewing, head bobbing, vibrissae twitching, scratching, face washing, “star gazing”; 3, partial-body clonus, occasional myoclonic jerks, shivering; 4, whole-body clonus, “corkscrew” turning and flipping, loss of posture, rearing, falling; 5, non-intermittent seizure activity; and 6, wild running, bouncing, tonic-clonic seizures.

### Post-status epilepticus model of epileptogenesis

Status epilepticus was induced by a single dose of pilocarpine hydrochloride (250 mg/kg) (Sigma Aldrich, St. Louis, MO) administered *i.p.* 30 minutes after methyl scopolamine nitrate (1 mg/kg; *i.p.*, Sigma Aldrich). Animals were placed in individual Plexiglass cages and monitored by laboratory personnel during and after SE. Seizures were rated according Racine’s score. Non-intermittent seizure activity, stages 3 and/or 4, for each mouse was limited to 90 minutes using a single dose of diazepam (10 mg/kg, *i.p.*, Sigma Aldrich). Each animal was monitored by trained laboratory personnel in a temperature-controlled surgical room until full locomotor recovery was observed (2–4 hours). Surviving animals (60%) were randomized by number assignment and placed in individual cages in an animal room with an artificial 12-hour light/dark cycle with access to food and water *ad libitum*.

### PAF-r antagonist administration

The anti-seizure activity of novel PAF-r antagonists (LAU-0901, LAU-09015, LAU-09017, LAU-09019, LAU-09018, LAU-09019, LAU-09020, LAU-09021, LAU-09023 and LAU-09025 (60 mg/kg/i.p.) in sterile Baxter 0.9% sodium chloride) was assessed by administration two hours prior to PTZ testing. Antagonist activity was compared to vehicle alone. In a second set of experiments, either 1 mL of vehicle or PAF-r antagonists (LAU-0901 or LAU-09021 60 mg/kg/i.p.) were administered at the same dosage daily for 5 days beginning 24 hours post-SE termination.

### Local field potential recordings and analysis

For local field potential (LFP) analysis, a silicon probe with 16 electrodes (spacing 100 μm, NeuroNexus, Ann Arbor, MI) was implanted in the right dorsal hippocampus of each mouse (from Bregma: 1.80 mm posterior; 1–1.5 mm lateral and 2.70 mm depth) 98 days after SE under anesthesia induced by a mixture of ketamine (200 mg/kg) and xylazine (10 mg/kg) (Vedco Inc., Saint Joseph, MO) using a surgical microscope and sterilized neurosurgical instruments. Briefly, during surgery the probe was placed on superficial layers of the cortex and then moved inward slowly with the aid of stereotaxic equipment (Kopf Instruments, Tujunga, CA). The resulting hole was covered by Surgicel (Ethicon Inc., San Angelo, TX) and saturated with a sterile cerebral spinal fluid (Harvard Apparatus, Holliston, MA). A stainless steel screw (Plastic One, Roanoke, VA) was implanted in the occipital bone as a ground wire for the silicon probe. Plastic One gel (Plastic One) was used to attach the probe and screw it to the skull. After recovery from anesthesia, mice were placed for recordings in individual Plexiglass cages and allowed to explore freely; food and water were provided *ad libitum*. LFP from the hippocampi were recorded from the headstage connected with the probe, amplified (1000×), band-pass filtered (0.1–300 Hz), and digitized at 1 KHz using MAP system data acquisition 7 days after surgery. Briefly, continuous LFP activity (4–5 minutes) from each freely-moving mouse was recorded and sampled (10–12 samples/hour) every 5 minutes from 10:00 a.m. to 4:00 p.m. using a MAP (Plexon) and video-recorder system for 5 consecutive days. LFPs were analyzed using NeuroExplorer (Next Tech Solutions, Inc., Austin, TX). Delta epochs (3–6 seconds each) from each LFP were determined by calculating the ratio of delta and theta frequency bands in the hippocampal CA1 region^70^, without artifacts or noise, by an investigator blinded to the treatment and confirmed during a period of immobility by visual inspection of video-LFP recordings. Band frequencies for delta (0.1–3.9 Hz), theta (4–8 Hz), beta (13–20 Hz), low gamma (21–40 Hz), and bands from 200–300 Hz were selected from the LFPs using power spectral density (PSD) analysis (NeuroExplorer, Next Technologies, Madison, AL)^14^. Then, average values of PSD of each band were compared from samples (n = 42 each group) of the PAF-r antagonist LAU-09021- and vehicle-treated animals. For automatic assessment of high frequency spikes the signal was filtered at 250–300 Hz band and analyzed using the offline sorter threshold function. Successive spike amplitude activity above the baseline was quantified using NeuroExplorer combined with offline sorter software. Artifacts such as head movement or grooming were excluded by visual inspection of video-LFP recordings. At the end of the experiment, verification of the probe placement in the dorsal hippocampus was confirmed by histology^13^ for anatomic-physiological correlation of hippocampal layers.

### Rapid kindling model of epileptogenesis

Bipolar electrode units (Plastic One Inc., Roanoke, VA, U.S.A.) were implanted in the dorsal right hippocampus (coordinates: 2.3 mm caudal to bregma; 1.75 mm lateral to midline; 2.00 mm ventral to dura) and ground wire was placed on the occipital bone, guided by stereotaxic procedure under anesthesia induced by intraperitoneal injection of a mixture of ketamine hydrochloride: xylazine (200 mg/kg: 10 mg/kg; Vedco). One week after surgery, kindling was achieved by sub-convulsive electrical stimulation at 30-min intervals (six stimulations per 10-s train containing 50-Hz biphasic pulses of 100-lA amplitude. To test the seizure susceptibility responses, sub-convulsive electrical stimulations (as during kindling) were given 1 week after kindling (rekindling).

### *In situ* hybridization

Mice were deeply anesthetized and brains were dissected according to previous procedures^17^, and a 385 bp fragment from the coding region of the PAF-r mRNA was amplified from mouse brain cDNA (Clonotec) by PCR and inserted into pCRrII TOPO (Invitrogen). ^35^S-labeled riboprobes were transcribed in sense and antisense directions.

### Liquid chromatography tandem mass spectrometry

PAF concentration in hippocampal samples was calculated as follows: brain enzymes were inactivated using high-powered microwave irradiation (10 KW, 400 V, 750 ms) focused on the head after anesthesia induced by isofluorane, followed by rapid immersion of the mouse head in ice water. Protein precipitates were separated by centrifugation, and solvent extracts were pre-equilibrated at pH 3.0 in 10% methanol/water, loaded to 500 mg C18 columns (Varian, Palo Alto, CA, U.S.A.), and then eluted with 1% methanol/ethyl acetate. Eluates were concentrated on an N_2_ stream evaporator. Samples were loaded to a liquid chromatograph-tandem mass spectrometer (LC-MS-MS; LC-TSQ Quantum, Thermo-Finnigan, Waltham, MA, U.S.A.) installed with a Biobasic-AX column (Thermo-Hypersil- Keystone, Bellefonte, PA, U.S.A.) (100 · 2.1 mm, 5-lm particle sizes). Samples were eluted in a linear gradient [100% solution A (40:60:0.01 methanol/water/acetic acid pH 4.5) to 100% solution B (99.99:0.01 methanol/acetic acid)] at a flow rate of 300 ll/min for 30 min. LC effluents were diverted to an electrospray-ionization probe (ESI) on a TSQ Quantum (Thermo-Finnigan) triple quadrupole mass spectrometer^13^; lipid standards (Cayman Chem., Ann Arbor, MI, U.S.A.) were used for tuning, optimization, and calibration curves. The instruments were set on full-scale mode to detect parent ions and also were set to selected-reaction mode (SRM) for quantitative analysis to detect product ions simultaneously. The selected parent/product ions (m/z) and collision energy (v) obtained on negative ion detection mode were 370.0/171.3/2 for PAF^17^.

### Dendrite spine detection and analysis

Brains were processed following established procedures according to the manufacturer’s instructions (FD Rapid GolgiStain Kit, FD Neurotechnologies, Inc., Columbia, MD). Coronal sections (40 μm) were made and then mounted, air-dried, dehydrated in alcohol, cleared in xylene and cover-slipped. Coronal sections were scanned using brightfield microscopy and magnified at 100X. In addition, individual areas were photographed at 40× and then apical and basal dendrites from Golgi-impregnated neurons were selected from treated mice using imaging application software (OlyVIA, Olympus, Center Valley, PA). Z-stacks (step size = 0.3 μm) of dendrites from the stratum oriens (OR), lacunosum-moleculare layers (L-M) of the CA1 and outer molecular layer (OM) from the dentate gyrus (DG) were captured using a 100×/oil objective. Images were recorded using an Axioplan 2 microscope (Carl Zeiss Inc., Thornwood, NY) coupled with AxioCam and Axiovision software (Carl Zeiss Inc.). Dendrite density and length of dendritic spines were quantified with Image J (National Institute of Health). A minimum of 10 dendrites were calculated per animal for the following regions: OR, L-M and DG. We then calculated the number of dendritic spines per segment of individual dendrites per hippocampal subfield for each treated group of animals.

### Statistics

The data retrieved from each experiment were averaged and expressed as mean ± S.E.M. For statistical significance, one time point (or more than two observations) was analyzed using Student’s *t*-Test and ANOVA followed by post-hoc tests (Tukey-Kramer and Hsu’MCB). Correlation analysis was performed for spine density (number of spines per 10 μm of dendrite segment cell and dendritic spine length) using Pearson’s correlation analysis. A *p-*value < 0.05 was considered significant. All data analyses, including sample size were conducted using JMP 8.0 statistical software from SAS (Cary, NC).

## Additional Information

**How to cite this article**: Musto, A. E. *et al*. Dysfunctional epileptic neuronal circuits and dysmorphic dendritic spines are mitigated by platelet-activating factor receptor antagonism. *Sci. Rep.* **6**, 30298; doi: 10.1038/srep30298 (2016).

## Supplementary Material

Supplementary Information
