# Corrigendum: Dysfunctional epileptic neuronal circuits and dysmorphic dendritic spines are mitigated by platelet-activating factor receptor antagonism

**DOI:** 10.1038/srep32253

**Published:** 2016-09-02

**Authors:** Alberto E. Musto, Robert F. Rosencrans, Chelsey P. Walker, Surjyadipta Bhattacharjee, Chittalsinh M. Raulji, Ludmila Belayev, Zhide Fang, William C. Gordon, Nicolas G. Bazan

Scientific Reports 6: Article number: 3029810.1038/srep30298; published online: 07 22 2016; updated: 09 02 2016

In this Article, reference 15 is duplicated as reference 13. The correct reference 15 appears below:

Bazan, N. G. Is there a molecular logic that sustains neuronal functional integrity and survival? Lipid signaling is necessary for neuroprotective neuronal transcriptional programs. *Mol. Neurobiol*. **50**, 1–5 (2014).

