# Supplementary material for: Dysfunctional epileptic neuronal circuits and dysmorphic dendritic spines are mitigated by platelet-activating factor receptor antagonism
Source: Sci Rep. 2016 Jul 22;6:30298. doi: 10.1038/srep30298 (PMC4957208; doi:10.1038/srep30298)

**Dysfunctional epileptic neuronal circuits and dysmorphic dendritic spines are mitigated by platelet-activating factor receptor antagonism**

**Running Title: PAF receptor antagonism limits aberrant neural connectivity**

**Authors**

Alberto E. Musto^1^, Robert F. Rosencrans^1^, Chelsey P. Walker^1^, Surjyadipta Bhattacharjee^1^, Chittalsinh M. Raulji^1,2^, Ludmilla Belayev^1^, Zhide Fang^3^, William C. Gordon^1^, Nicolas G. Bazan^1^*.

**Addresses**

^1^Neuroscience Center of Excellence, Louisiana State University Health Sciences Center, 2020 Gravier Street, 8^th^ floor, New Orleans, Louisiana 70112, USA

^2^Department of Pediatrics, Hematology-Oncology, Louisiana State University Health Sciences Center and Children’s Hospital of New Orleans, New Orleans, Louisiana 70118, USA

^3^Biostatistics, School of Public Health, Louisiana State University Health Sciences Center, 2020 Gravier Street, New Orleans, Louisiana 70112, USA

**Corresponding Author**

Address correspondence to: Nicolas G. Bazan, Neuroscience Center of Excellence, School of Medicine, Louisiana State University Health Sciences Center, 2020 Gravier Street, New Orleans, LA 70112, USA. Phone: 504-599-0831; Fax: 504-568-5801; E-mail: [nbazan@lsuhsc.edu](mailto:nbazan@lsuhsc.edu)

**Supplementary Figure 1.**

Representative Racine’s scores from individual PAF-r ^+/+^ and PAF-r^-/-^ mice under high pentylenetetrazol (PTZ) dosages. PAF-r deficient mice require excess PTZ to induce seizures, as compared to PAF-r ^+/+^ mice. At 110 mg/kg i.p. PTZ, blinded reviewers score a PAF-r^-/-^ mouse at a Racine’s score of 1, whereas its wild type counterparts achieve a range of higher seizure intensities. At 185 mg/kg i.p. PTZ PAF-r^-/-^ mice reach the seizure intensities achieved by wild type counterparts at lower doses. Locomotor seizures were video recorded and quantified according Racine’s score and classified as follows: 0, normal behavior—walking, exploring, sniffing, grooming; 1, immobile, staring, jumpy, curled-up posture; 2, automatisms—repetitive blinking, chewing, head bobbing, vibrissae twitching, scratching, face washing, “star gazing”; 3, partial-body clonus, occasional myoclonic jerks, shivering; 4, whole-body clonus, “corkscrew” turning and flipping, loss of posture, rearing, falling; 5, non-intermittent seizure activity; and 6, wild running, bouncing, tonic-clonic seizures.

**Supplementary Figure 2.**

PAF-r antagonist compounds attenuate seizure susceptibility. (a) Single dose of PAF-r antagonist compounds (60 mg/kg; ip) or vehicle (0.1 mL of sterile 0.9% sodium chloride, Baxter ip) was administered two hours before a single dose of pentylenetetrazol (PTZ) (35 mg/kg; ip). Data represents average and bars ±S.E.M: LAU-0901 p<0.031 others: p<0.0001 at 60’ compared to vehicle (VEH), ANOVA; Seizures were scored according to Racine’s score. (b) Molecular structure of new PAF antagonist compounds.

**Supplementary Figure 3.**

Assessment for seizure susceptibility and hippocampal hyper-excitability after status epilepticus induced by pilocarpine**.** Representative seizure susceptibility and hippocampal hyper-excitability after pilocaprine induces post status epilepticus (epilepsy). Local field potential (LFP) activity of hippocampal CA1 region and its respective spectrogram (below) showing epileptiform discharge after intraperitoneal administration of sub-convulsive dose of pentylenetetrazol (35mg/kg) in naïve mouse (a) and mouse 110 days twenty four hours after SE. Note the short latency to stage 2 and subsequent progression to stage 5 followed by death in epileptic mouse.

**Supplementary Figure 4.**

Spine lengths at 105 days post-status epilepticus from control (n=5), vehicle-treated (n=5), and LAU 09021-treated (n=4) mice across three hippocampal regions. No significant differences are observed. Spine lengths were assessed from 60 μm thick, Golgi stained coronal sections, imaged at 100x, and measured using ImageJ. Bars indicate averages. Error bars represent SEM.

Supplementary Figure 1


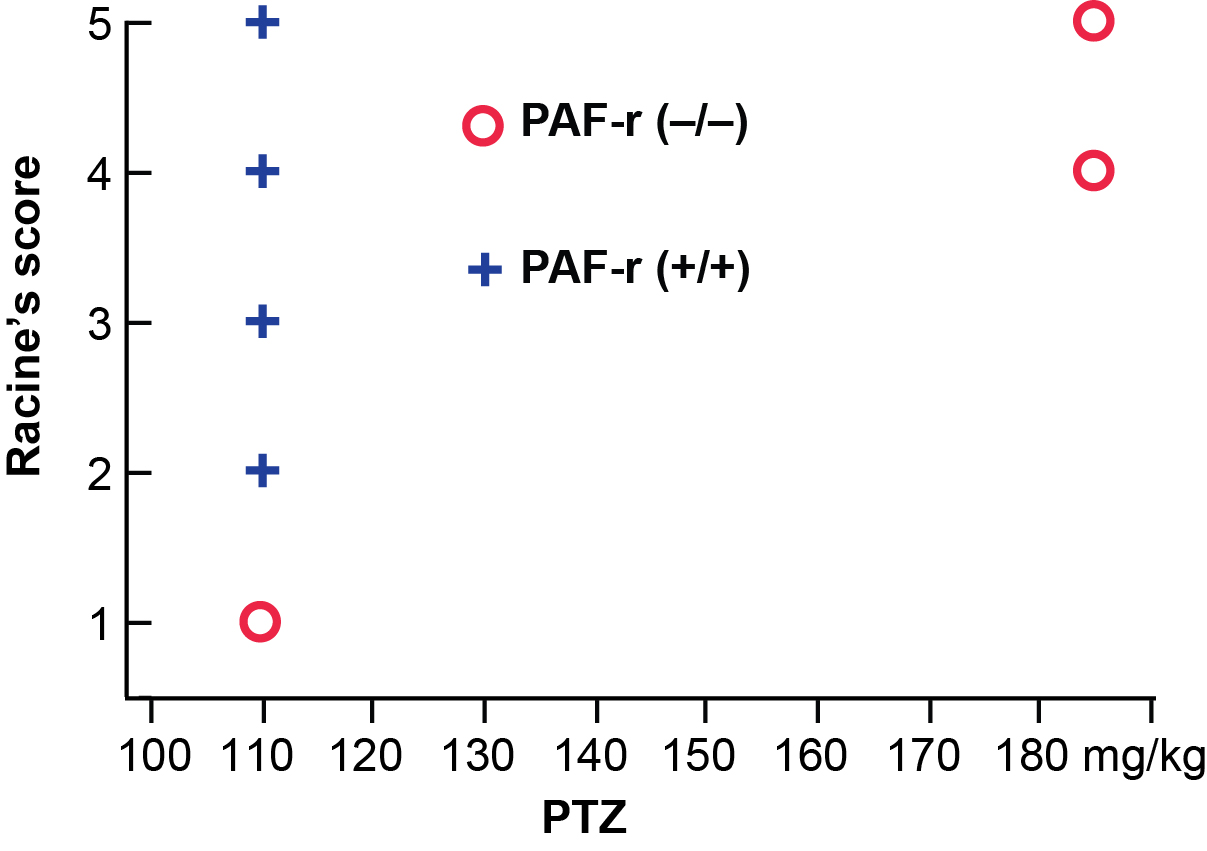


Supplementary Figure 2


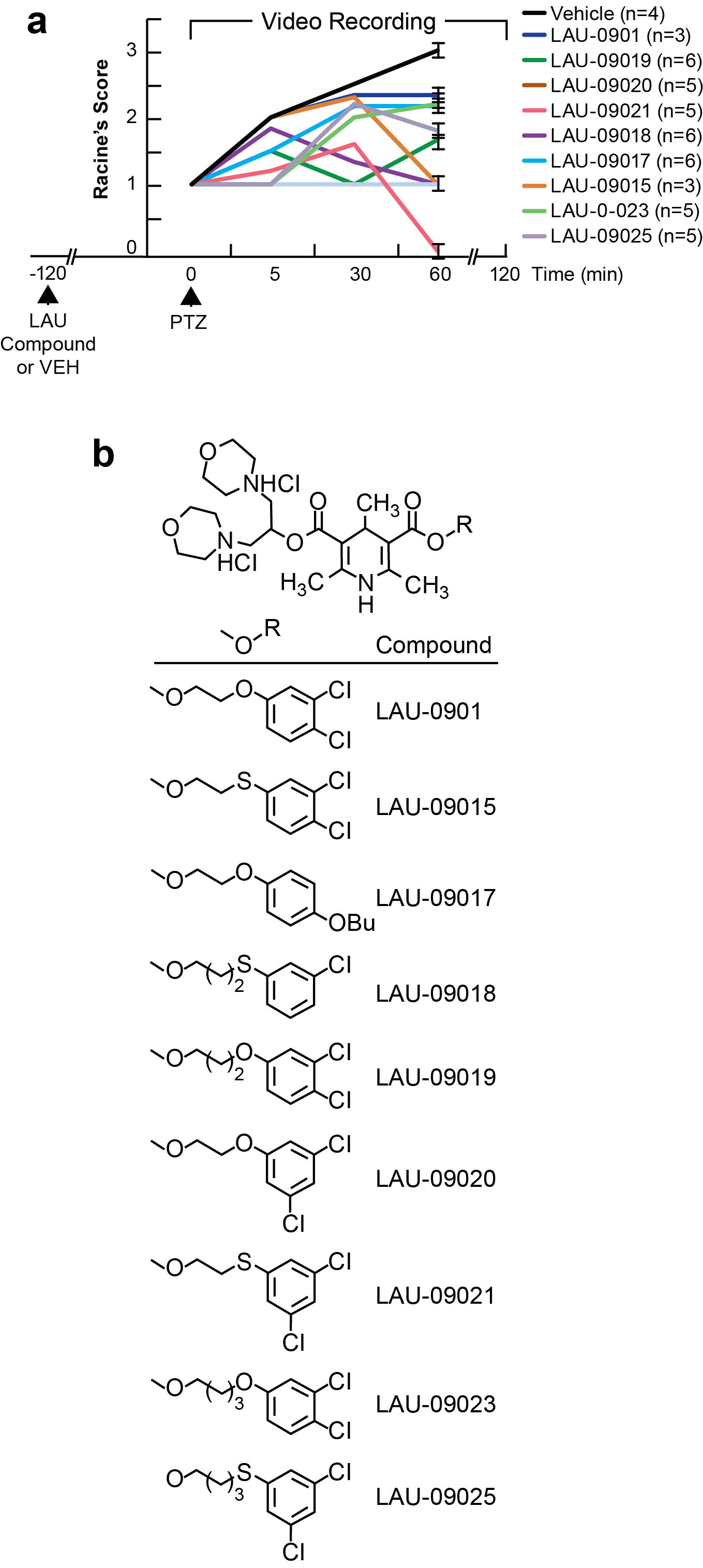


Supplementary Figure 3


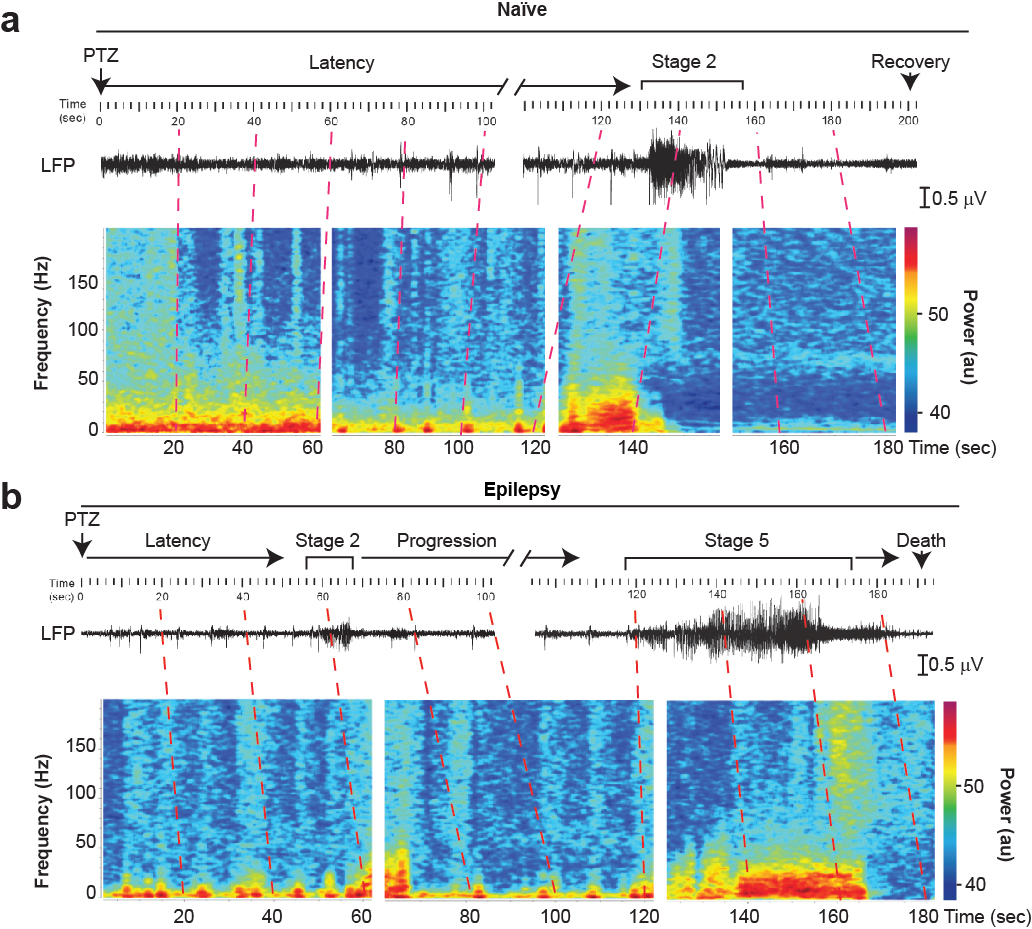


Supplementary Figure 4


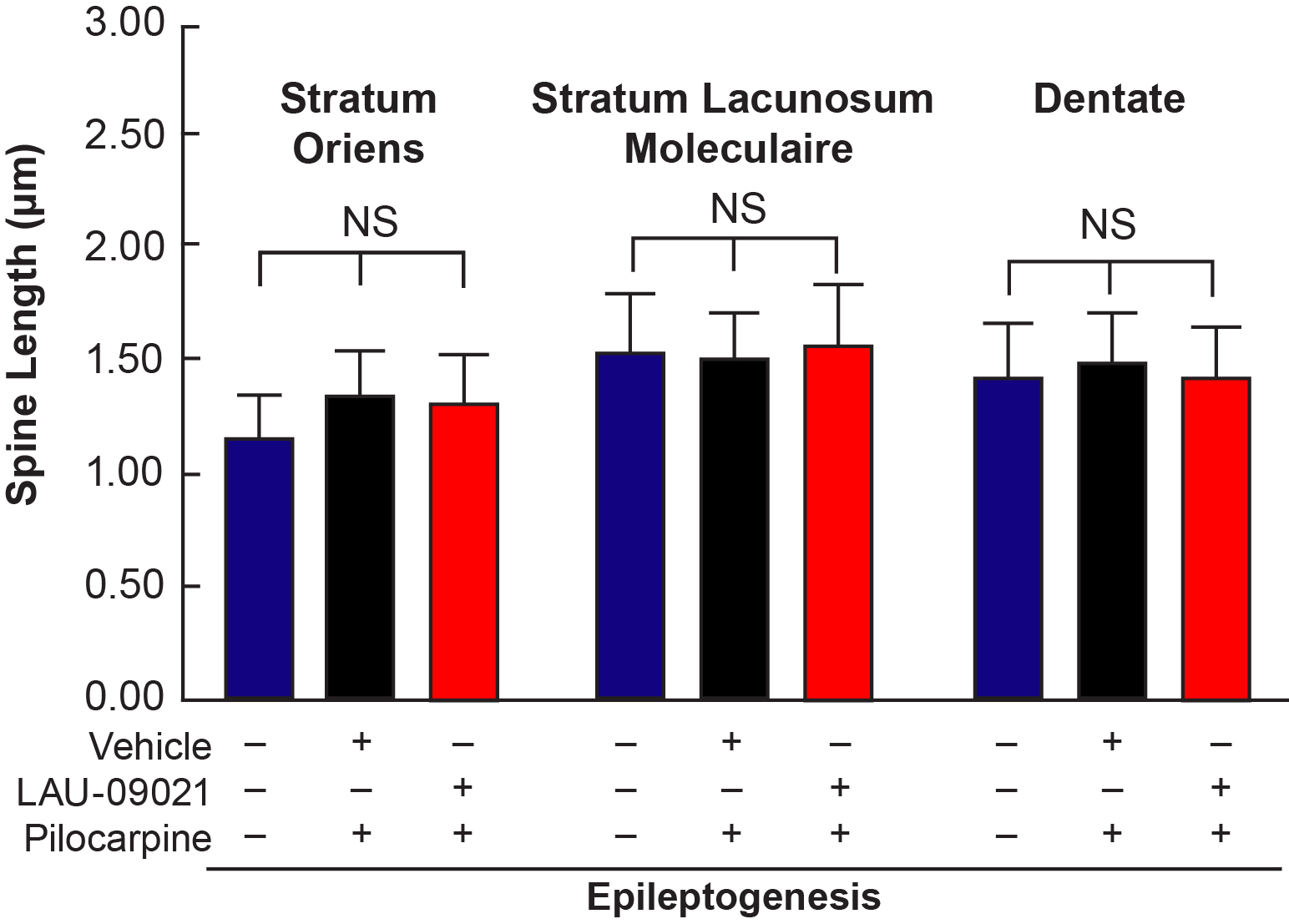

Supplement: Supplementary Information [file srep30298-s1.doc]
